# The Use of Bedside Ultrasound in the Evaluation of Patients Presenting with Signs and Symptoms of Pulmonary Embolism

**DOI:** 10.1155/2013/312632

**Published:** 2013-09-19

**Authors:** Adarsh N. Patel, L. Connor Nickels, F. Eike Flach, Giuliano De Portu, Latha Ganti

**Affiliations:** Department of Emergency Medicine and Center for Brain Injury Research and Education, University of Florida College of Medicine, 1329 SW 16th Street, P.O. Box 100186, Gainesville, FL 32610-0186, USA

## Abstract

Evaluation of patients that present to the emergency department with concerns for the diagnosis of pulmonary embolism can be difficult. Modalities including computerized tomography (CT) of the chest, pulmonary angiography, and ventilation perfusion scans can expose patients to large quantities of radiation especially if the study has to be repeated due to poor quality. This is particularly a concern in the pregnant population that has an increased incidence of pulmonary embolism and may not be able to undergo multiple radiographic studies due to fetal radiation exposure. This paper presents a case of a pregnant patient with signs and symptoms concerning pulmonary embolism. The paper discusses the use of bedside ultrasound in the evaluation of patients with pulmonary embolism.

## 1. Case Presentation

A 20-year-old G2P1 pregnant female at 22 weeks from her last menstrual period presents to the emergency department as a transfer patient from an outside hospital. She was evaluated for two days of progressively worse shortness of breath. The major concern at the outside hospital was a pulmonary embolism. They performed a chest CT scan that was reported as inconclusive for pulmonary embolism secondary to poor quality, and thus she was transferred for further evaluation of pulmonary embolism. 

Upon arrival to the ED, the patient denied any personal or family history of DVT, pulmonary embolism, or clotting disorders. Her only identifiable risk factor for pulmonary embolism was her pregnancy. On physical examination the patient was well appearing and oriented to person, place, and time. She was clearly tachypneic with a heart rate of 120–140 s bpm. The rest of her vital signs and physical examination were normal. An EKG was performed in the emergency department which showed sinus tachycardia without S1Q3T3 sign. 

**Figure 1:**
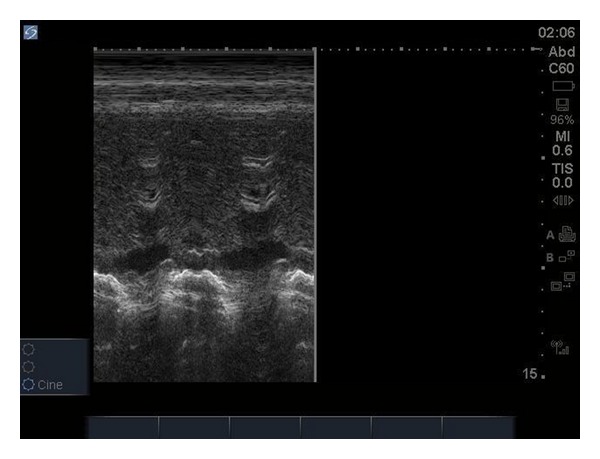
M-mode on IVC showing normal IVC collapse on respiration.

**Figure 2:**
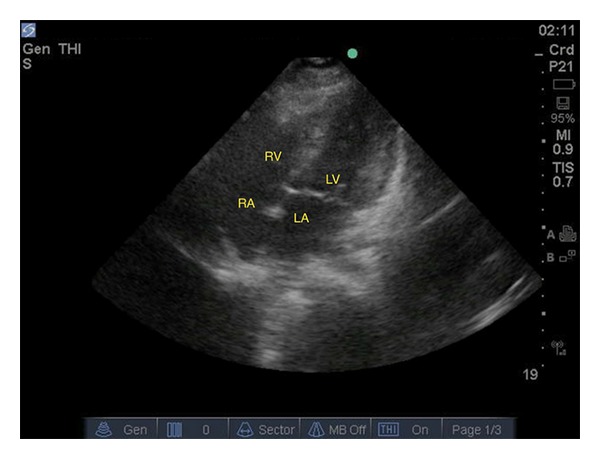
Apical four-chamber view showing normal RV : LV ratio.

Ultrasound evaluation in the emergency department was performed with the focus on evaluation of pulmonary embolism. A 2–4 MHz phased-array probe was used to perform the echocardiogram. A subxiphoid view of the heart was performed, and no pericardial effusion or wall motion abnormalities were noted. The IVC diameter was not dilated and had normal variation with respirations (Figure 1). A parasternal short axis view at the level of the pulmonary artery was performed and did not show any free-floating thrombus in either the right heart or pulmonary artery. The parasternal short axis view at the level of the papillary muscles did not show any flattening or bowing of the intraventricular septum into the left ventricle. No right ventricular dilation was noted (Figure 2). In total, besides the tachycardia there were no other findings concerning pulmonary embolism on ultrasound. CT chest for pulmonary embolism was repeated and this time an adequate quality film was obtained and was negative for pulmonary embolism. 

Upon further history taking, the patient admitted to be ingesting 1-2 tablets of aspirin every 4 hours on a regular basis for tooth pain. Her arterial blood gas showed a mixed metabolic acidosis and respiratory alkalosis. The history and lab work raised high suspicion for salicylate intoxication and thus a salicylate level was drawn. The salicylate level came back elevated, and the patient was diagnosed with salicylate toxicity. She was admitted and treated appropriately. She was discharged 4 days later in stable condition. 

## 2. Discussion

The use of bedside ultrasound evaluation of patients presenting to the emergency department with signs and symptoms of pulmonary embolism has been controversial. Rapid diagnosis using ultrasound can lead to earlier preparation of thrombolytic administration especially in the critical ill patients where rapidity is crucial [1]. There are multiple sonographic findings that support the diagnosis of acute pulmonary embolism and can be broken down into two major categories: direct and indirect signs. Direct signs included visualization of a free-floating thrombus in the right heart or pulmonary artery [1, 2]. Indirect signs include right ventricular dilation (RV/LV ratio >0.6–1 : 1), flattening or bowing of the intraventricular septum into the left ventricle, right ventricular systolic dysfunction, McConnell's sign, and IVC dilation without inspiratory collapse [2–5]. 

Direct signs of pulmonary embolism are best visualized in the parasternal short axis at the level of the pulmonary artery. Free-floating right ventricular thrombi are highly specific for pulmonary embolism but uncommonly occur. The prevalence of right ventricular thrombi in patients with pulmonary embolism is only 4 to 18% [6]. Right ventricular thrombus may not be diagnostically useful due to its poor sensitivity; however, it is prognostically valuable and indicates a high mortality rate of 44.7% [7]. Multiple case reports have been published with the utility of transthoracic echo (TTE) in the visualization of massive or saddle pulmonary emboli [1, 8]. Limitation of using transthoracic ultrasound includes visualization of only the proximal main, right, and left pulmonary arteries. Emboli distal to this region would be missed on TTE and thus transesophageal echocardiogram or helical CT may need to be performed [5, 9].

Right ventricular (RV) dilation may be seen in patients with pulmonary embolism especially if the embolus is massive and causing right heart strain. Right ventricular dilation has a poor sensitivity of only 31% and a high specificity of 94% [10]. Right ventricular dilation may lead to flattening/bowing of the intraventricular septum (IVS) into the left ventricle and lead to eventual right ventricular dysfunction. RV dilation and IVS flattening are best visualized in the parasternal short axis at the level of the papillary muscles. With IVS flattening the left ventricle loses its “O” shaped appearance and becomes “D” shaped [2]. Right ventricular dysfunction, as defined by tricuspid regurgitation velocity greater than 2.9 m/s, has been shown to have a sensitivity of 51% and a specificity of 88% [10]. Another definition of right ventricular dysfunction includes RV end-diastolic diameter >30 mm in the precordial view, hypokinesis of the free wall, and dilation of the right pulmonary artery [11–13]. Patients with pulmonary embolism and right ventricular dysfunction as evidenced on ultrasound have been shown to have higher mortality rates 0–7% versus 14–18% [3, 14].

McConnell's sign is akinesia of the mid-free wall of the right ventricle but normal motion at the apex. The best view to assess for McConnell's sign is the apical four-chamber view. McConnell's sign is used to differentiate patients with an acute PE from those with pulmonary hypertension secondary to other causes such as idiopathic pulmonary hypertension, chronic obstructive pulmonary disease, obstructive sleep apnea, and right-sided myocardial infarction [5]. McConnell's sign has been shown to have a sensitivity of 77%, specificity of 94%, positive predictive value of 71%, and a negative predictive value of 96% [4]. IVC dilation with loss of inspiratory collapse has been shown to correlate to patients presenting with pulmonary embolism [3, 5, 9].

## 3. Conclusion

Bedside ultrasound in the evaluation of pulmonary embolism has become a useful tool among emergency medicine physicians. It allows for rapid evaluation in time-sensitive critically ill patients and thus can promote prompt treatment without having to delay for further radiography studies. Bedside ultrasound also has a role amongst pregnant patients that may not be able to undergo multiple radiographic studies due to large amounts of fetal radiation exposure. The specificity of sonographic evidence of pulmonary embolism has been shown to be high; however, its poor sensitivity implies that further testing may be necessary in order to fully rule out a pulmonary embolism.
